# The multitarget fecal immunochemical test versus the fecal immunochemical test for programmatic colorectal cancer screening: a cross-sectional intervention study with paired design

**DOI:** 10.1186/s12885-022-10372-2

**Published:** 2022-12-12

**Authors:** P. H. A. Wisse, W. de Klaver, F. van Wifferen, L. Meiqari, M. Bierkens, M. J. E. Greuter, B. Carvalho, M. E. van Leerdam, M. C. W. Spaander, E. Dekker, V. M. H. Coupé, M. de Wit, G. A. Meijer

**Affiliations:** 1 Department of Pathology, Netherlands Cancer Institute, Plesmanlaan 121, Amsterdam, CX the Netherlands; 2 Department of Gastroenterology and Hepatology, Erasmus University Medical Center, Doctor Molewaterplein 40, Rotterdam, GD 3015 the Netherlands; 3 Department of Gastroenterology and Hepatology, Amsterdam University Medical Centers, Location University of Amsterdam, Meibergdreef 9, Amsterdam, AZ 1105 the Netherlands; 4 Department of Epidemiology and Data Science, Amsterdam University Medical Centers, Location Vrije Universiteit, De Boelelaan 1117, Amsterdam, HV 1081 the Netherlands; 5 Department of Gastro-intestinal Oncology, Netherlands Cancer Institute, Plesmanlaan 121, Amsterdam, CX 1066 the Netherlands

**Keywords:** Fecal immunochemical test, Multitarget fecal immunochemical test, Screening, Advanced neoplasia, Colorectal cancer, Health economic modelling

## Abstract

**Background:**

Many screening programs for colorectal cancer (CRC) use the fecal immunochemical test (FIT) to triage individuals for colonoscopy. Although these programs reduce CRC incidence and CRC-related mortality, the detection of advanced precursor lesions (advanced adenomas and advanced serrated polyps) by FIT could be improved. As an alternative for FIT, the antibody-based multitargetFIT (mtFIT) has been proposed. The mtFIT measures three protein markers: hemoglobin, calprotectin, and serpin family F member 2. In a retrospective diagnostic accuracy study in a large colonoscopy-controlled series (*n* = 1284), mtFIT showed increased sensitivity for advanced neoplasia (AN), at equal specificity, compared to FIT (42.9% versus 37.3%; *p* = 0.025). This increase was mainly due to a higher sensitivity of mtFIT for advanced adenomas (37.8% versus 28.1% for FIT; *p* = 0.006). The present mtFIT study aims to prospectively validate these findings in the context of the Dutch national CRC screening program.

**Method:**

The mtFIT study is a cross-sectional intervention study with a paired design. Eligible subjects for the Dutch FIT-based national CRC screening program are invited to perform mtFIT in addition to FIT. Samples are collected at home, from the same bowel movement, and are shipped to a central laboratory by postal mail. If either one or both tests are positive, participants are referred for colonoscopy. Detailed colonoscopy and pathology data are centrally stored in a national screening database (ScreenIT; Topicus, Deventer, the Netherlands) that is managed by the screening organization, and will be retrieved for this study. We aim to determine the relative sensitivity for AN, comprising of CRC, advanced adenomas and advanced serrated polyps, of mtFIT compared to FIT at an equal positivity rate. Additionally, we will use the Adenoma and Serrated Pathway to Colorectal CAncer model to predict lifetime health effects and costs for programmatic mtFIT- versus FIT-based screening. The target sample size is 13,131 participants.

**Discussion:**

The outcome of this study will inform on the comparative clinical utility of mtFIT versus FIT in the Dutch national CRC screening program and is an important step forward in the development of a new non-invasive stool test for CRC screening.

**Trial registration:**

Clinicaltrials.gov; NCT05314309, registered April 6th 2022, first inclusions March 25th 2022 https://clinicaltrials.gov/ct2/results?cond=&term=NCT05314309&cntry=&state=&city=&dist=.

## Background

Colorectal cancer (CRC) is responsible for approximately 10% of all cancer cases and related deaths, making it a highly prevalent and deadly disease. In 2020, worldwide around 1.9 million new CRC cases and over 900.000 CRC-related deaths occurred [1]. CRC survival is inversely related to CRC stage at diagnosis, which underlines the importance of early detection through screening. The gradual transition from normal epithelium through an adenoma or serrated polyp stage to CRC provides an opportunity for prevention and early detection [2–5]. Indeed, CRC screening programs, introduced over the last decade, have reduced CRC incidence and CRC-related mortality [6–10].

In line with the World Health Organization's recommendations, many countries have implemented national CRC screening programs based on a fecal immunochemical test (FIT), which detects hemoglobin in stool, followed by colonoscopy for those testing positive. Advantages of FIT-based screening programs are the high participation rate, the limited burden for participants, and the efficient use of colonoscopy resources [11–13]. Even though FIT-based screening effectively reduces CRC incidence and CRC-related mortality, there still is substantial room for improvement, especially in accurately detecting advanced precursor lesions (advanced adenomas and advanced serrated polyps) [14, 15]. Detecting high-risk lesions at a precursor stage, before their progression to cancer, allows for less complicated removal of those lesions during colonoscopy without the risk of metastasis, resulting in better survival [16]. However, a higher detection rate should not come at the cost of specificity. Therefore, there is a need for cost-effective, non-invasive screening tests that outperform FIT in the detection of advanced neoplasia (AN), at an equal positivity rate, to improve the performance of current CRC screening programs [17, 18].

While many stool biomarkers for CRC screening have been investigated, only a few tests have made it to implementation [19–21]. The most well-known example is the multitarget stool DNA (mt-sDNA) test, which is widely used in the United States [22]. However, the mt-sDNA test is not considered an alternative for FIT in many countries with programmatic CRC screening, based on cost-effectiveness and logistical considerations, as the mt-sDNA test is more costly than FIT and based on whole stool samples instead of small stool samples.

As an alternative, over the past decade, we have developed a multitarget fecal immunochemical test (mtFIT) that could be suitable for programmatic CRC screening [12, 23, 24]. This mtFIT consists of antibody-based assays for a combination of three protein biomarkers (hemoglobin, calprotectin and serpin family F member 2), and can be performed on small stool samples, which are collected using the same collection devices as for regular FIT-based screening. Recently, we have demonstrated in a retrospective diagnostic accuracy study with 1284 participants that, at an equal specificity of 96.6%, the sensitivity of mtFIT for AN was higher than that of FIT (42.9% versus 37.3%, respectively; *p* = 0.025). This increase was mainly due to a 35% increase in advanced adenoma detection (37.8% versus 28.1% for mtFIT and FIT, respectively; *p* = 0.006) [12]. Early health technology assessment, evaluating the potential of implementing the mtFIT in a CRC screening program, indicated that mtFIT-based screening could lead to a reduction of 12% in CRC incidence and 8% in CRC-related mortality compared to FIT-based screening. Moreover, it showed that mtFIT-based screening could be cost-effective compared to FIT-based screening. As a next step, we now set out to validate these findings in a cross-sectional intervention study with a paired design in an intended-use population, i.e. participants of the Dutch national CRC screening program.

### Objectives

#### Primary objective

The primary objective of this study is to assess the relative sensitivity (calculated as the relative detection rate) of mtFIT compared to FIT for AN at an equal positivity rate in a head-to-head comparison in an intended-use population. AN comprises CRC, advanced adenomas and advanced serrated polyps.

#### Secondary objectives

A secondary objective is to assess the relative sensitivity for CRC, advanced adenomas, and advanced serrated polyps after one round of screening with mtFIT or FIT. In addition, we aim to assess the long-term cost-effectiveness of mtFIT- versus FIT-based programmatic CRC screening.

## Methods/Design

### Study design

The mtFIT study is a cross-sectional intervention study with a paired design comparing mtFIT to FIT. Participants will take two samples from the same bowel movement. One of the laboratories appointed to analyze FIT samples collected during the Dutch national CRC screening program will also analyze the mtFIT samples. If either or both tests are positive, participants are referred for colonoscopy. All colonoscopy and pathology data are collected and centrally stored in a national screening database (ScreenIT; Topicus, Deventer, The Netherlands), managed by the screening organization, and will be retrieved for this study. As recommended by the Dutch Health Council, the ethical review and approval of the study were issued by the Dutch Ministry of Health, Welfare and Sport in April 2020. The study is registered in ClinicalTrials.gov (NCT05314309) [25]. The current mtFIT study is being conducted with the FAIR principles in mind: to have the data be Findable, Accessible, Interoperable and Reusable.

### Study population

The mtFIT study is conducted in an intended-use population [18, 26]. To this end, participants are randomly selected from the target population of the Dutch national CRC screening program (*n* = 2.000.000 annually). The screening organization will send invitations until 13,131 participants are included. Inclusion criteria for the mtFIT study are equal to those of the Dutch national CRC screening program. Dutch residents aged 55 to 75 years old are eligible, except for those [1] undergoing treatment for CRC, or [2] having had a colonoscopy less than 5 years ago, or [3] undergoing colonoscopy surveillance because of another gastrointestinal disease or [4] because of an increased risk of CRC due to a hereditary or familial CRC syndrome. In order for subjects to be able to read the participant information and give informed consent, they need to have a sufficient understanding of the Dutch language.

### Sample size calculation

The sample size is based on the McNemar test for testing the difference in detection rates between mtFIT and FIT in a paired design. The calculation used data reported by the screening organization for 2020, with a FIT detection rate for AN of 1.2%, and assumed that mtFIT would result in a 20% increased detection rate of 1.44%. Then, under the assumption of an 80% overlap in AN detection, a two-sided significance level of 5%, and a power of 90% to detect a 20% increased detection rate, a total number of 13,131 participants is required.

### Subject recruitment and informed consent

The screening organization, responsible for executing the Dutch national CRC screening program, selects and recruits subjects. A computer-run algorithm selects a random sample from the participants of the Dutch national CRC screening program (SPSS, version 23, IBM Corp, Armonk, NY). Each subject has an unique study code used in all correspondence. An invitation is sent to subjects four weeks prior to their planned regular screening invitation, informing them about the study and giving them the opportunity to participate. This invitation includes an information brochure, an informed consent form, and a link to the study website which contains an animation video explaining the study, and all study brochures (in Dutch) [27]. Individuals participating in the study have to sign the informed consent form and send it to the Netherlands Cancer Institute, where the informed consent is stored following the European General Data Protection Regulation. In addition to requesting informed consent for participation in the study, participants are also asked for permission to re-use obtained data and material for future research into early detection of CRC, including the storage of collected stool samples in a biobank and keeping residual material obtained during a possible colonoscopy.

### Sample collection

**Fig. 1:**
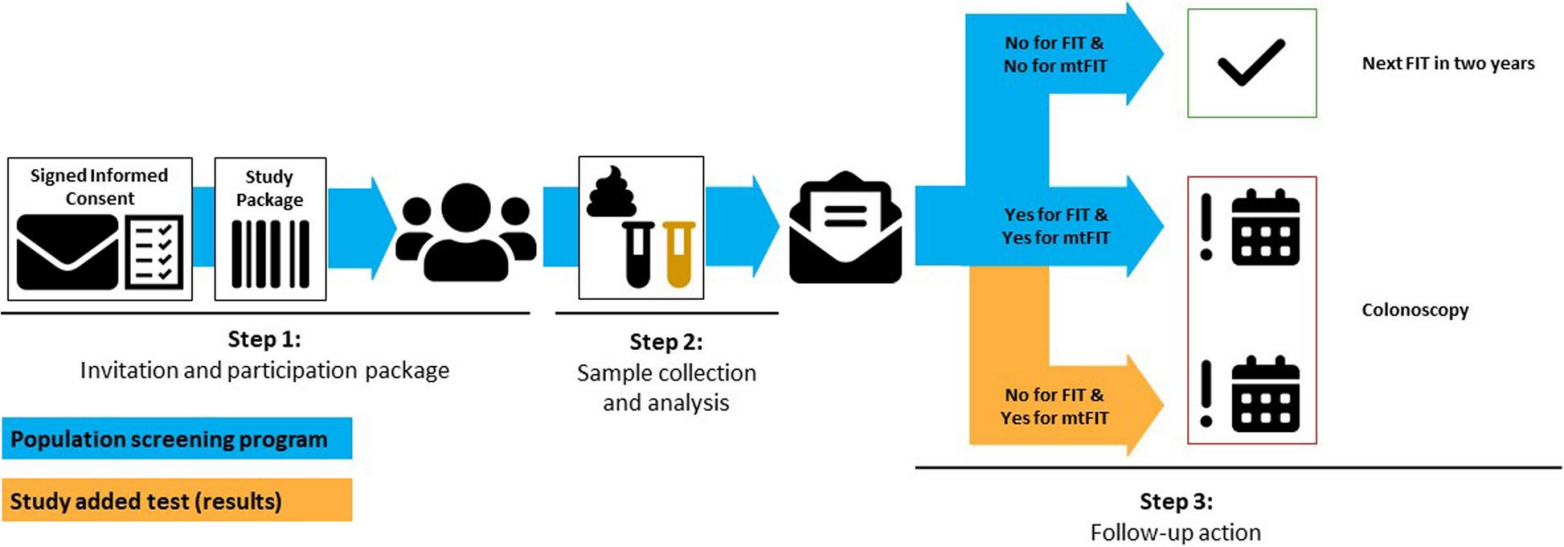
Flow-scheme of the cross-sectional intervention study. Step 1; After invitation and written consent, participants will receive a study package at home with two tests (FIT and mtFIT). Step 2; Participants will perform the two tests at home and send them per post to the central laboratory where the tests are analyzed. Step 3; Analysis results will be communicated to the participant per post. In case either one or both tests have a positive result, then the participant will be referred for colonoscopy, otherwise they will be re-invited for screening 2 years later

Approximately four to eight weeks after their study invitation, at the moment of their planned invitation for the Dutch national CRC screening program, participants receive a participation package. This package includes an information brochure on the national CRC screening program and the mtFIT study, instructions for collecting the stool samples for the two tests, two collection tubes, a plastic sealing bag for safely shipping the collection tubes, and a return envelope (Fig. 1, step 1). The FOB-Gold (Sentinel, Milan, Italy) collection tube is used for FIT and the OC-Sensor (Eiken Chemica Co., Tokyo, Japan) for mtFIT. After completing the stool collection, the samples are returned to the laboratory by postal mail. Except for the extra collection tube and associated information, the study package is similar to the package used in the Dutch national CRC screening program.

### Laboratory procedures

The laboratory checks the returned envelopes for the presence of both FIT and mtFIT. When a participant returns just one of two collection tubes, he/she will be excluded from the study. If only the FIT is returned the FIT is analysed for the regular CRC screening program. In the laboratory, the two collection tubes follow separate workflows.

FIT samples will be analyzed according to the standard operating procedures of the Dutch national CRC screening program, using a fully-automated clinical chemistry analyzer (Bio Majesty JCA, DiaSys Diagnostic Systems, Holzheim, Germany). Quantitative hemoglobin test results for every sample are automatically communicated to the screening organization and stored in the ScreenIT database (Fig. 1, step 2). The used FIT cut-off for positivity is 47 μg hemoglobin / g feces and is similar to the cut-off used in the regular Dutch national CRC screening program [28].

For mtFIT, the assay, developed using the Meso Scale Discovery (MSD) platform, measures the hemoglobin, calprotectin, and serpin family F member 2 proteins with tailored antibody assays, using electrochemiluminescence [29]. The study setup allows for the analysis of 37 samples (in duplo) per run, next to multiple controls. For each protein, the mean of the duplicate values is computed and fed into the previously described mtFIT algorithm [12]. The mtFIT result is then communicated to the screening organization and stored in the ScreenIT database (Fig. 1, step 2).

### Final test result

Within 10 days of the arrival of the sample at the laboratory, the screening organization informs the participant of the final positive or negative stool test result. The result is positive if FIT and/or mtFIT are positive and negative if both FIT and mtFIT are negative (Fig. 1, step 3). For positive results, both the participant and the endoscopist are blinded for which test(s) was (were) positive. If a participant wants to know which test(s) was (were) positive before the colonoscopy, the participant will be excluded from the study.

### Clinical procedures

**Fig. 2:**
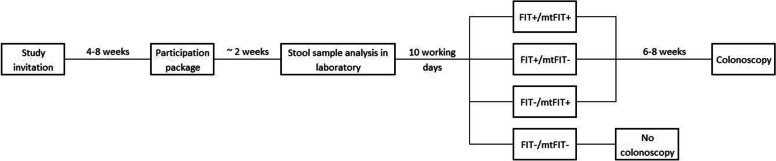
Participant timeline. Estimated periods of waiting time between the different steps of the study per participant

In the event of a positive stool test result, participants are referred for colonoscopy (Fig. 1, step 3). All lesions detected during colonoscopy are removed and sent for pathological evaluation. The quality of the colonoscopy and pathology examinations is controlled in the Dutch national CRC screening program, including certification of the centers and the individual endoscopists [30]. This includes structured reporting of both colonoscopy and pathology data in ScreenIT [31, 32]. The data of study participants will be made available to the study team in a pseudonymized form. AN is defined as the presence of a CRC, an advanced adenoma or an advanced serrated polyp. CRC stage is defined based on the American Joint Committee on Cancer TNM classification system. Advanced adenomas are defined as adenomas with a size ≥10 mm and/or high-grade dysplasia and/or a villous component (e.g. tubulovillous or villous adenoma). Advanced serrated polyps are serrated polyps with a size ≥10 mm and/or with any grade of dysplasia (Fig. 2).

### Data collection, management and monitoring

To define data management processes and workflow, the research team has developed standard operating procedures that follow the Good Clinical Practice guidelines. Study data are compiled and stored in the web-based electronic data capture system ‘Castor EDC’ (Ciwit B.V., Amsterdam, the Netherlands). Informed consent and participants’ data are pseudonymized by the screening organization, using unique identification codes. Each sample tube, either mtFIT or FIT, has a unique code with which the laboratory reports the mtFIT results to the study team. Only the screening organization has access to information that may reveal a participants’ identity.

For data sharing and transfer of the various data types obtained during this study, a secure cloud storage called Surfdrive (SURF, Utrecht, the Netherlands) is used that complies with the Dutch and European privacy legislation [33]. Access to the cloud storage is restricted to study staff, including the researchers, screening organization and laboratory, and granted at folder level.

Data monitoring is performed regularly to check completeness of data entry and warnings from the validation rules. Incidents regarding data and study logistics are documented and reviewed with the screening organization and the National Institute of Public Health and the Environment.

The study team aims to ensure that end-of-study integrated data complies with the FAIR principles using the cBioPortal platform hosted for Dutch institutions through Health-RI [34].

### Data analysis

Since colonoscopy data will only be available for participants with a positive FIT and/or mtFIT, absolute sensitivities of both tests cannot be determined. Therefore, the performance of mtFIT will be compared to FIT by determining the relative sensitivity (calculated as the relative detection rate) for AN as well as CRC, advanced adenomas and advanced serrated polyps, respectively. This comparison will be performed at an equal positivity rate.

### Health economic modelling

The externally validated Adenoma and Serrated pathway to Colorectal Cancer (ASCCA) model will be used to assess the long-term cost-effectiveness of programmatic mtFIT- versus FIT-based screening [35]. We will first set up the model to simulate the current Dutch national CRC screening program, which consists of biennial FIT-based screening between the age of 55 and 75 years. Participation to FIT and colonoscopy will be set to 73 and 92%, respectively, following the observed participation rates in the Dutch national CRC screening program. Subsequently, we will set up the model to simulate mtFIT-based screening, assuming the same screening protocol and participation rates as for FIT-based screening. The sensitivities of FIT for separate lesion types (i.e., CRC, advanced adenomas, advanced serrated polyps, non-advanced adenomas, non-advanced serrated polyps) that are currently used in the ASCCA model will be adapted with the relative sensitivities of mtFIT compared to FIT at an equal positivity rate to obtain absolute sensitivities of mtFIT. Outcomes of each screening strategy will include the number of CRC cases and deaths, number of colonoscopies, quality adjusted life-years and costs. The mtFIT-based screening will be compared to FIT-based screening by calculating CRC incidence and CRC-related mortality reductions as well as the incremental cost-effectiveness ratio (ICER) (the difference in costs divided by the difference in quality adjusted life-years). In addition, a threshold analysis will be performed to determine the maximal costs of the mtFIT at which mtFIT-based screening will be cost-effective compared to FIT-based screening assuming a willingness-to-pay threshold of €45.874 corresponding to the Dutch gross domestic product per capita in 2020 [36, 37].

## Discussion

Worldwide, CRC poses a heavy burden for both individuals affected by the disease as well as healthcare systems. Early detection of CRC by programmatic CRC screening has proven to be the most effective method to address this challenge [17]. Many countries have substantially invested in deploying such programs, that require extensive logistics. Consequently, any improvements of FIT-based screening will be much more easily adopted when the investments in these CRC screening programs can be re-used.

The opportunity to conduct this study in 13,131 participants of the Dutch national CRC screening program has major advantages. Screening trials require large numbers of participants and are inherently costly, while support from industry for screening studies like this one lags far behind what is common practice in drug development. The ability to benefit from the logistics, quality assurance, data management, and collection of colonoscopy and pathology outcome data is a tremendous facilitator, and provides the optimal scenario for evaluating mtFIT in an intended-use population. An inherent limitation is that the study is not fully colonoscopy controlled, since only participants with a positive stool test result will be referred for colonoscopy. Nevertheless, using a paired design still allows for determining the relative sensitivity (relative detection rate at equal positivity rate) of mtFIT for AN compared to FIT. The validation of new screening tests is recommended in an intended use population, by comaring the new screening test to an existing screening test with well-known impact on CRC-incidence and CRC-related mortality [18]. In addition, the study results will be used to model the long-term impact of mtFIT- versus FIT-based screening on CRC incidence and CRC-related mortality and to assess whether the mtFIT could replace FIT in programmatic CRC screening programs in a cost-effective way.

The assay technology used in the mtFIT is industry standard and allows multiple proteins to be analyzed simultaneously. However, the method requires substantial hands-on time in the laboratory, making it, at the moment, less suitable for use at the scale required for programmatic CRC screening programs. Nevertheless, this study is important in proving the clinical utility of the mtFIT combination of protein markers. The technical details of the platform ultimately used, pending the results of the mtFIT study, will depend on the choices made by any industry partner developing mtFIT into a commercial product.

In conclusion, mtFIT holds great potential for improving current FIT-based CRC screening programs while at the same time being compatible with the logistics of these screening programs, which strengthens the perspectives for implementation of the test. Next to validating earlier retrospective data, the planned study in the intended-use population will also provide a solid basis for determining the conditions under which mtFIT-based screening will be cost-effective compared to FIT-based screening. Thus, this study marks an important step forward in the development of a new stool-based test for programmatic CRC screening.

## Data Availability

The study data will become available after publication in a scientific journal on request from G.A. Meijer.

## Abbreviations

AN: Advanced neoplasia
ASCCA: Adenoma and Serrated pathway to Colorectal CAncer
CRC: Colorectal cancer
FIT: Fecal immunochemical test
mtFIT: Multitarget fecal immunochemical test
mt-sDNA test: The multitarget stool DNA test

## References

[1] Sung H, Ferlay J, Siegel RL, Laversanne M, Soerjomataram I, Jemal A (2021). Global Cancer Statistics 2020: GLOBOCAN Estimates of Incidence and Mortality Worldwide for 36 Cancers in 185 Countries. CA Cancer J Clin.

[2] Winawer SJ, Zauber AG (2002). The advanced adenoma as the primary target of screening. Gastrointest Endosc Clin N Am.

[3] Stryker SJ, Wolff BG, Culp CE, Libbe SD, Ilstrup DM, MacCarty RL (1987). Natural history of untreated colonic polyps. Gastroenterology..

[4] Pickhardt PJ, Kim DH, Pooler BD, Hinshaw JL, Barlow D, Jensen D (2013). Assessment of volumetric growth rates of small colorectal polyps with CT colonography: a longitudinal study of natural history. Lancet Oncol.

[5] O'Connell B, Hafiz N, Crockett S (2017). The Serrated Polyp Pathway: Is It Time to Alter Surveillance Guidelines?. Curr Gastroenterol Rep.

[6] Rosello S, Simon S, Cervantes A (2019). Programmed colorectal cancer screening decreases incidence and mortality. Transl Gastroenterol Hepatol.

[7] Ibanez-Sanz G, Mila N, Vidal C, Rocamora J, Moreno V, Sanz-Pamplona R (2021). Positive impact of a faecal-based screening programme on colorectal cancer mortality risk. PLoS One.

[8] Gini A, Jansen EEL, Zielonke N, Meester RGS, Senore C, Anttila A (2020). Impact of colorectal cancer screening on cancer-specific mortality in Europe: A systematic review. Eur J Cancer.

[9] Guo F, Chen C, Holleczek B, Schottker B, Hoffmeister M, Brenner H (2021). Strong Reduction of Colorectal Cancer Incidence and Mortality After Screening Colonoscopy: Prospective Cohort Study From Germany. Am J Gastroenterol.

[10] Bucchi L, Mancini S, Baldacchini F, Ravaioli A, Giuliani O, Vattiato R, et al. How a faecal immunochemical test screening programme changes annual colorectal cancer incidence rates: an Italian intention-to-screen study. Br J Cancer. 2022. 10.1038/s41416-022-01813-7 PMC9345854 35444286

[11] Joseph DA, Meester RG, Zauber AG, Manninen DL, Winges L, Dong FB (2016). Colorectal cancer screening: Estimated future colonoscopy need and current volume and capacity. Cancer..

[12] de Klaver W, Wisse PHA, van Wifferen F, Bosch LJW, Jimenez CR, van der Hulst RWM (2021). Clinical Validation of a Multitarget Fecal Immunochemical Test for Colorectal Cancer Screening : A Diagnostic Test Accuracy Study. Ann Intern Med.

[13] Zhong GC, Sun WP, Wan L, Hu JJ, Hao FB (2020). Efficacy and cost-effectiveness of fecal immunochemical test versus colonoscopy in colorectal cancer screening: a systematic review and meta-analysis. Gastrointest Endosc.

[14] Mendelsohn RB, Winawer SJ, Ahnen DJ (2020). Incidence of Colorectal Cancer Matters. Gastroenterology..

[15] Grobbee EJ, Wisse PH, Schreuders EH, van Roon A, van Dam L, Zauber AG (2022). Guaiac-based faecal occult blood tests versus faecal immunochemical tests for colorectal cancer screening in average-risk individuals. Cochrane Database Syst Rev.

[16] Kaltenbach T, Anderson JC, Burke CA, Dominitz JA, Gupta S, Lieberman D (2020). Endoscopic Removal of Colorectal Lesions-Recommendations by the US Multi-Society Task Force on Colorectal Cancer. Gastroenterology..

[17] Shaukat A, Levin TR. Current and future colorectal cancer screening strategies. Nat Rev Gastroenterol Hepatol. 2022. 10.1038/s41575-022-00612-y PMC9063618 35505243

[18] Young GP, Senore C, Mandel JS, Allison JE, Atkin WS, Benamouzig R (2016). Recommendations for a step-wise comparative approach to the evaluation of new screening tests for colorectal cancer. Cancer..

[19] Bosch LJ, Carvalho B, Fijneman RJ, Jimenez CR, Pinedo HM, van Engeland M (2011). Molecular tests for colorectal cancer screening. Clin Colorectal Cancer.

[20] Issa IA, Noureddine M (2017). Colorectal cancer screening: An updated review of the available options. World J Gastroenterol.

[21] Rasmussen SL, Krarup HB, Sunesen KG, Pedersen IS, Madsen PH, Thorlacius-Ussing O (2016). Hypermethylated DNA as a biomarker for colorectal cancer: a systematic review. Color Dis.

[22] Imperiale TF, Ransohoff DF, Itzkowitz SH, Levin TR, Lavin P, Lidgard GP (2014). Multitarget stool DNA testing for colorectal-cancer screening. N Engl J Med.

[23] Bosch LJ, de Wit M, Pham TV, Coupé VM, Hiemstra AC, Piersma SR (2017). Novel Stool-Based Protein Biomarkers for Improved Colorectal Cancer Screening: A Case–Control Study. Ann Intern Med.

[24] Dominitz JA (2021). A Tailored FIT for Improved Colorectal Cancer Screening. Ann Intern Med.

[25] Prospective Clinical Validation of a Novel Multitarget FIT in CRC Screening (mtFIT) [Internet]. ClinicalTrials.gov. [cited June 30, 2022]. Available from: https://www.clinicaltrials.gov/ct2/home.

[26] Doubeni CA, Lau YK, Lin JS, Pennello GA, Carlson RW (2022). Development and evaluation of safety and effectiveness of novel cancer screening tests for routine clinical use with applications to multicancer detection technologies. Cancer..

[27] Wetenschappelijk onderzoek naar vroege opsporing van darmkanker (multitargetFIT): Antoni van Leeuwenhoek hospital; [Available from: https://www.avl.nl/alles-over-kanker/informatie-over-klinische-studies-trials/multitargetfit/#uitleg.

[28] Toes-Zoutendijk E, van Leerdam ME, Dekker E, van Hees F, Penning C, Nagtegaal I (2017). Real-time monitoring of results during first year of Dutch colorectal cancer screening program and optimization by altering fecal immunochemical test cut-off levels. Gastroenterology..

[29] Debad JDGE, Leland JK (2004). Clinical and biological applications of ECL.

[30] Bronzwaer MES, Depla A, van Lelyveld N, Spanier BWM, Oosterhout YH, van Leerdam ME (2019). Quality assurance of colonoscopy within the Dutch national colorectal cancer screening program. Gastrointest Endosc.

[31] Casparie M, Tiebosch AT, Burger G, Blauwgeers H, van de Pol A, van Krieken JH (2007). Pathology databanking and biobanking in The Netherlands, a central role for PALGA, the nationwide histopathology and cytopathology data network and archive. Cell Oncol.

[32] van Doorn SC, van Vliet J, Fockens P, Dekker E (2014). A novel colonoscopy reporting system enabling quality assurance. Endoscopy..

[33] Teeuwen N. Secure data storage: SURFdrive; 2021 [updated 15-04-2022. Available from: https://wiki.surfnet.nl/display/SURFdrive/Secure+data+storage.

[34] Wilkinson MD, Dumontier M, Aalbersberg IJ, Appleton G, Axton M, Baak A (2016). The FAIR Guiding Principles for scientific data management and stewardship. Sci Data.

[35] Greuter MJ, Xu XM, Lew JB, Dekker E, Kuipers EJ, Canfell K (2014). Modeling the Adenoma and Serrated pathway to Colorectal CAncer (ASCCA). Risk analysis : an official publication of the Society for Risk Analysis.

[36] Hutubessy R, Chisholm D, Edejer TT (2003). Generalized cost-effectiveness analysis for national-level priority-setting in the health sector. Cost Eff Resour Alloc.

[37] Regionale kerncijfers; nationale rekeningen: CBS Statline; [updated November 19, 2022. Available from: https://opendata.cbs.nl/#/CBS/nl/dataset/84432NED/table.

